# Pseudo tumor of the urinary bladder in a child

**DOI:** 10.4103/0974-7796.62910

**Published:** 2010

**Authors:** LI Gonzalez-Granado

**Affiliations:** Paediatrics Hospital, 12 octubre, Carretera Andalucía S/N, Madrid, Spain

Sir,

I have read with great interest the article by Srivastava *et al*. 'Pseudotumor of the urinary bladder in a child: A case report and review of the literature' published in the last issue of the journal.[1] The surgical information provided is very helpful, but I would like to make some comments about the clinical and microbiological aspects of the case (and the review of the literature).

It has been shown that the presence of mycobacterial infection (*Mycobacterium tuberculosis* or *M. avium intracellulare*) is a rare - but not as infrequent as previously thought - coexistence with this tumor. There are reports of lung, larynx, lymph nodes and even skin mycobacterial infection associated with myofibroblastic tumor.[2,3] Urine should be collected in order to rule out tuberculosis infection, which is a possible diagnosis in patients with inflammatory pseudotumor. The diagnosis is confirmed by identification of acid-fast bacilli. New culture techniques and molecular biology methods such as polymerase chain reaction allow early identification of Mycobacterium tuberculosis.[4]

An eight-year-old child suffering from urinary tuberculosis should be treated for six months with anti-mycobacterial drugs, but he also needs evaluation to rule out immunodeficiency (especially Mendelian susceptibility to mycobacterial infection. This means testing the interferon-gamma-IL12 pathway).[5]
